# Supplementary material for: Characterization of the hemodynamic response function in white matter tracts for event-related fMRI
Source: Nat Commun. 2019 Mar 8;10:1140. doi: 10.1038/s41467-019-09076-2 (PMC6408456; doi:10.1038/s41467-019-09076-2)
Supplement: Supplementary file 1 — Supplementary Information [file 41467_2019_9076_MOESM1_ESM.pdf]

Supplementary Information for

Characterization of the hemodynamic response function in white matter tracts for event-related fMRI

Li et al.

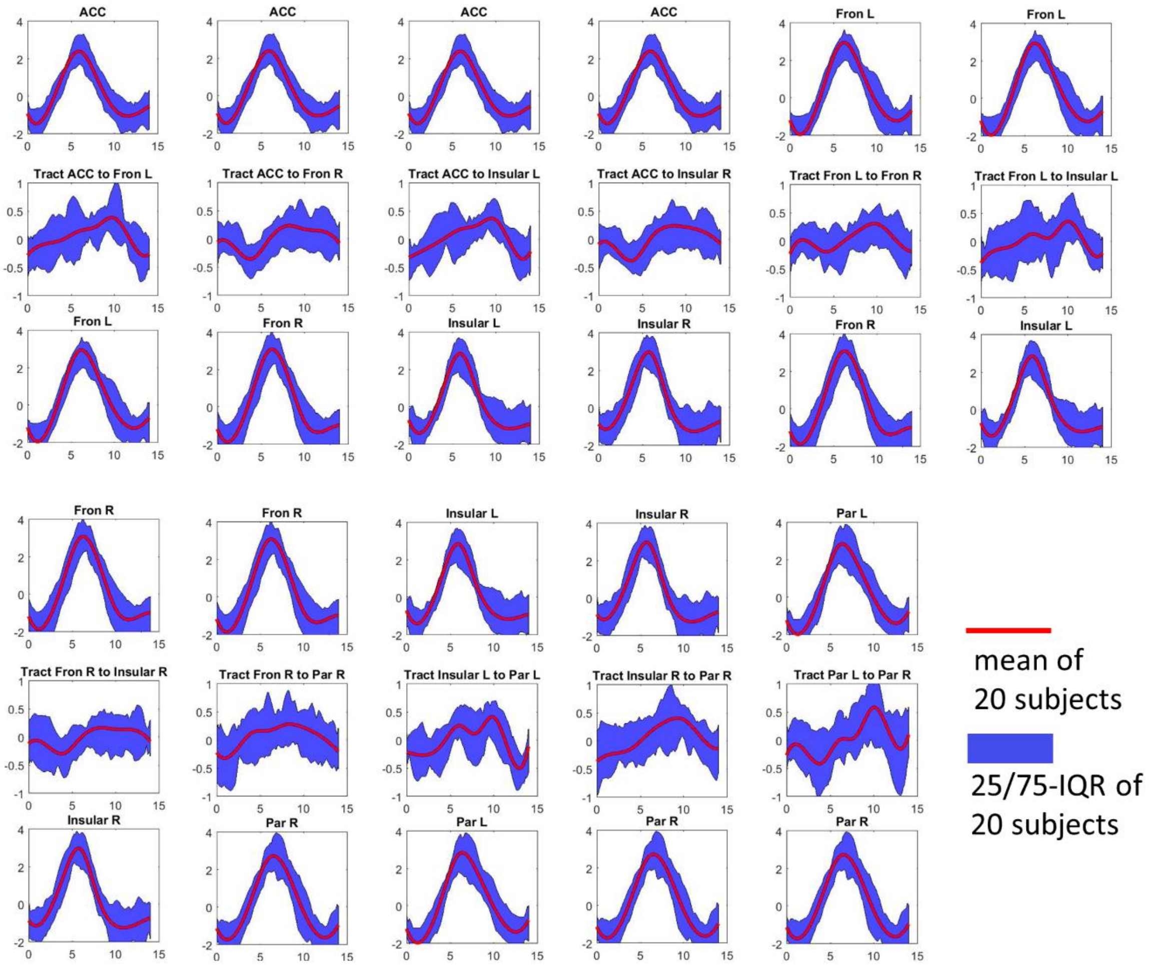

Supplementary Fig. 1. Average time course in WM tracts and their connecting GM clusters obtained by simply averaging the time courses in the epochs following each incongruent event. The time course of a WM tract was displayed in the center of each panel with the time course of its connecting GM clusters vertically aligned at the top and bottom. *x*-axis is the time (seconds), *y*-axis is the signal intensity (a.u.). Time=0 is aligned to the onset of each incongruent stimulus. IQR = Interquartile Range. Source data are provided as a Source Data file.

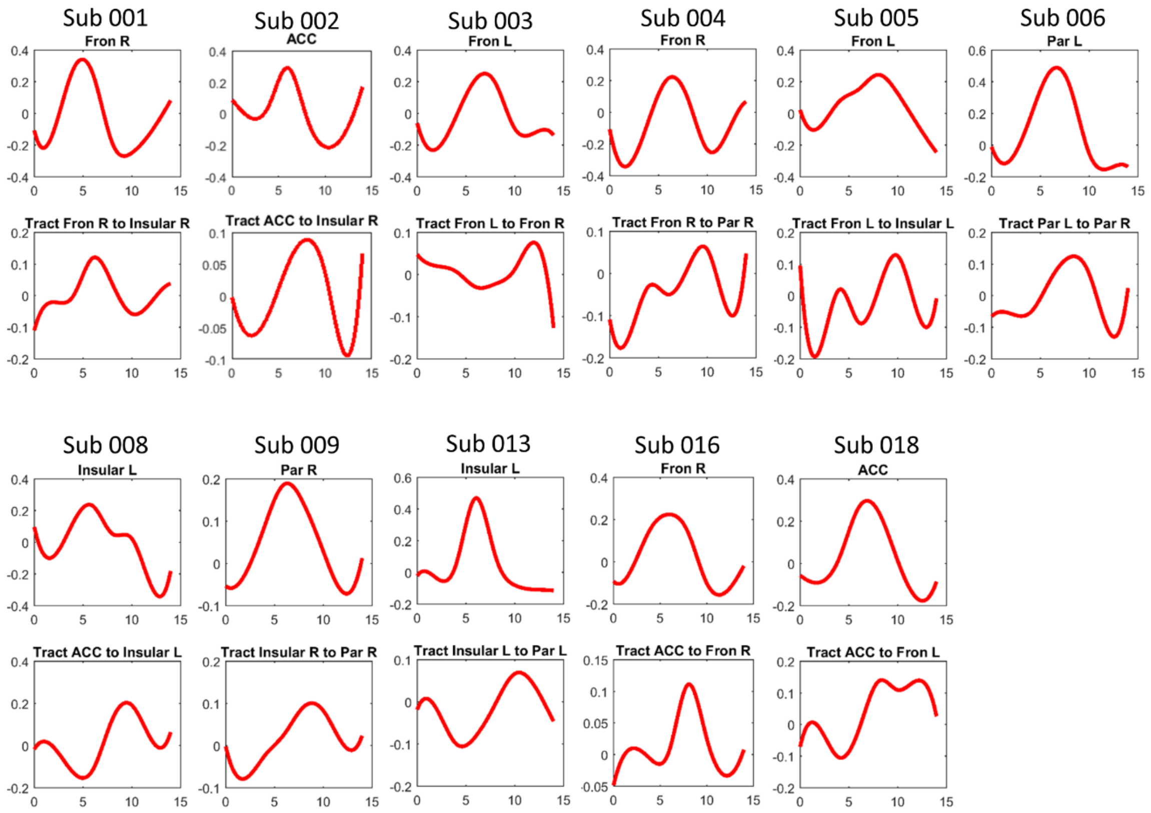

Supplementary Fig. 2. White matter time courses of eleven WM tracts in selected subjects. Source data are provided as a Source Data file.

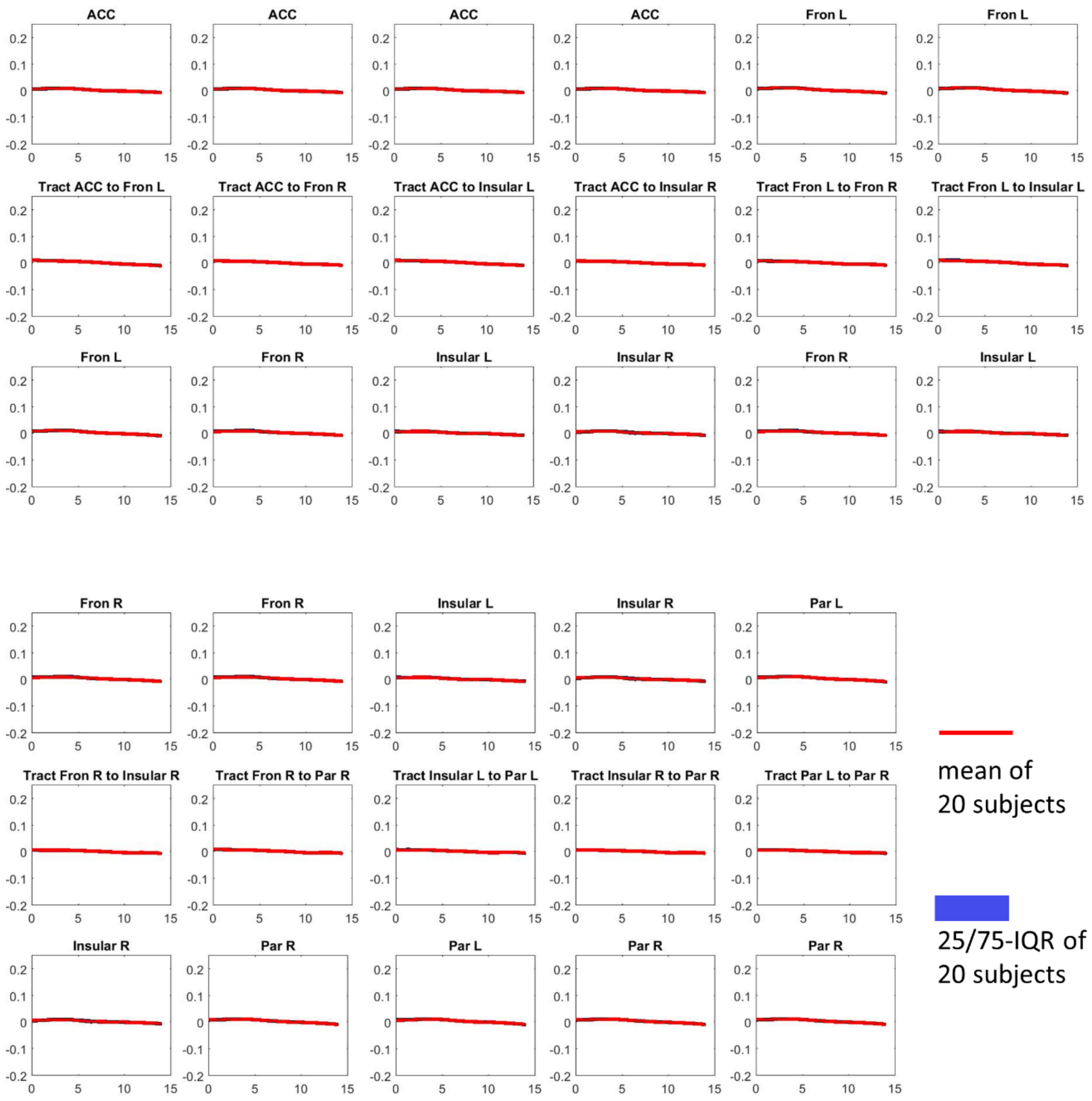

Supplementary Fig. 3. Event-unrelated time course in WM tracts and their connected GM clusters. The time course of a WM tract was displayed in the center of each panel with the time course of its connecting GM clusters vertically aligned at the top and bottom. *x*-axis is the time (seconds), *y*-axis is the signal intensity (a.u.). Time=0 is aligned to the onsets of random selected stimuli. IQR = Interquartile Range. Source data are provided as a Source Data file.

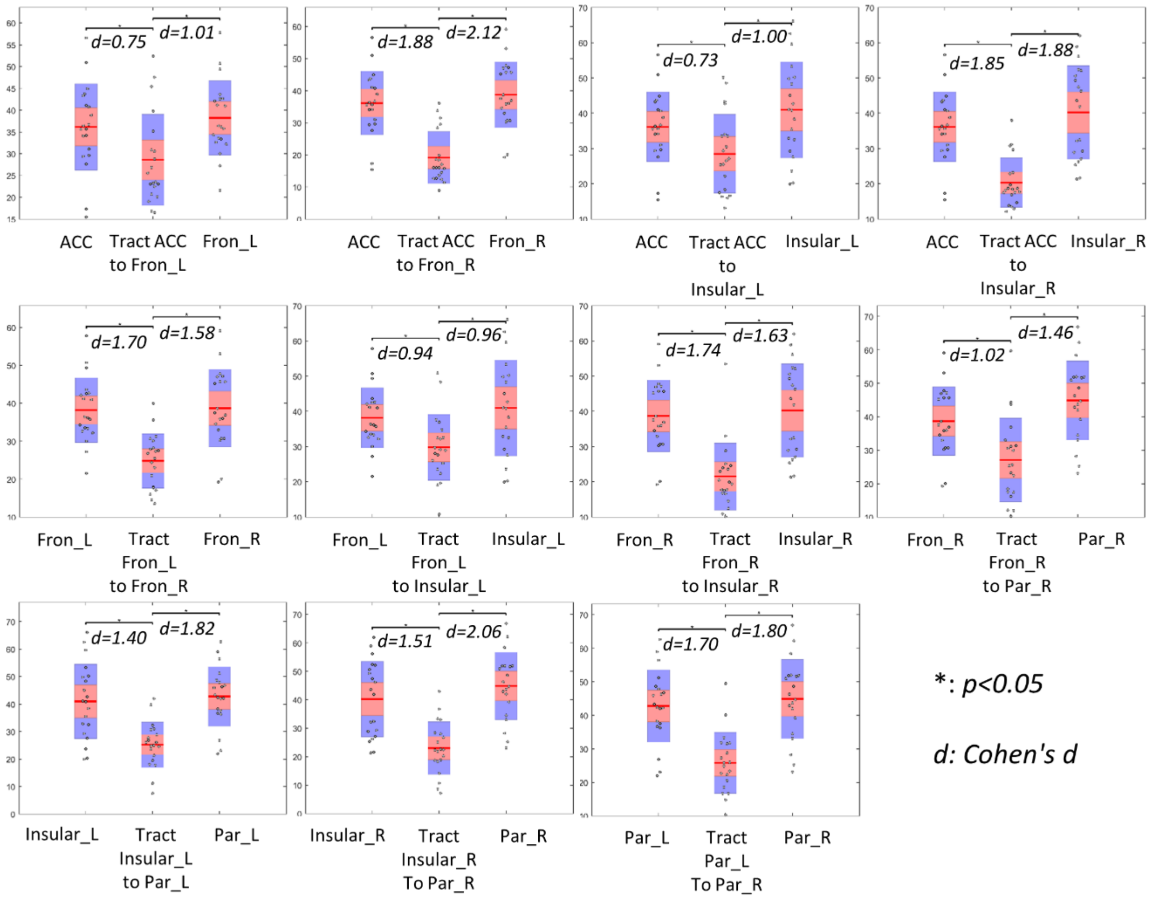

Supplementary Fig. 4. Average AUC of WM tracts and their connected GM clusters. Average AUC of one WM tract was displayed in the center of each panel with the average AUC of its connecting GM clusters on either side. *y*-axis is the AUC (a.u.). Points are laid over a 1.96 SEM (95% confidence interval) in red, a 1 SD in blue and a center line that denotes the median. All WM tracts showed significant decreased AUC ($P<0.05$, t-test) in comparison with their connecting GM clusters. Source data are provided as a Source Data file.

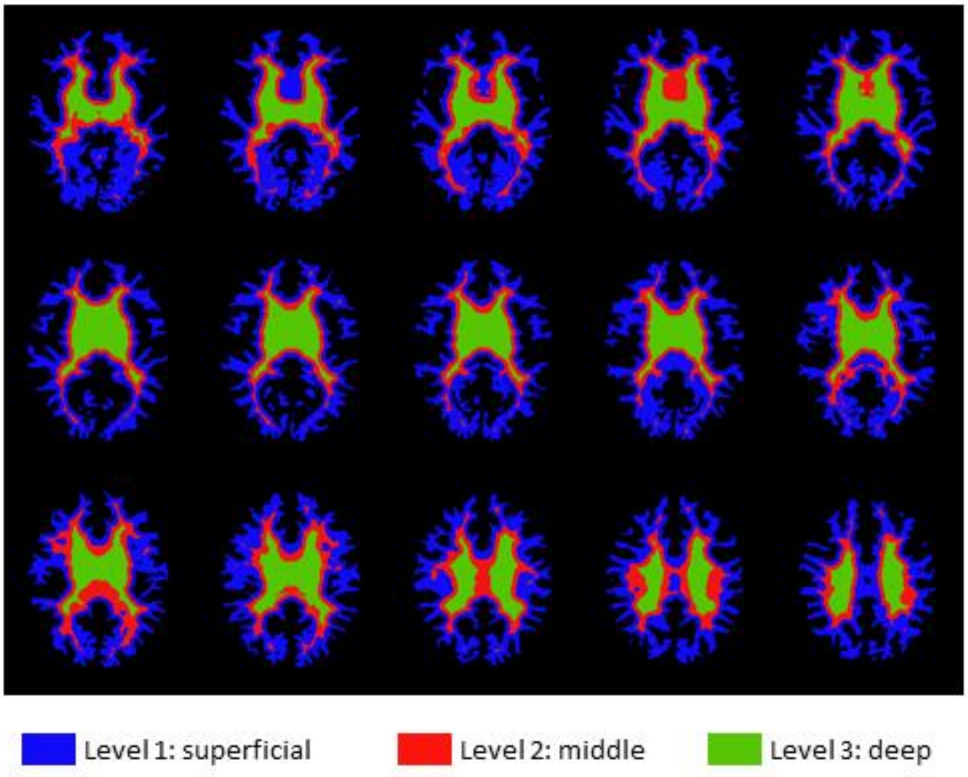

Supplementary Fig. 5. Three levels of WM masks, including superficial, middle and deep WM, created by successive erosion of original WM mask.

Supplementary Table 1. Reproducibility of tractography between pairs of activated clusters in 20 subjects. Red: Zero fiber or false positive fibers can be reconstructed from 20 subjects. Orange: Fibers can be reconstructed from some subjects. Green: Fibers can be reconstructed from all 20 subjects. 11 of 21 possible tracts were reproducible across all subjects. The remaining 10 tracts that showed poorer reproducibility were excluded from the subsequent time-course analysis. The tractography approach failed to retrieve the pathways between clusters that were bilaterally asymmetric (e.g., Fron_L and Par_R) in most subjects. False positive reconstructions (e.g. between Fron_R and Insular_L) could be observed in one or two subjects where the fibers propagated through ventricles or the longitudinal fissure, which failed to represent anatomically plausible pathways. The commissural fibers between bilateral insular cortices, though symmetric in anatomy, could not be reconstructed in all subjects. In addition, the association fibers connecting Fron_L and Par_L could be reconstructed in more than half of the subjects but were still considered to have insufficient representation of the population.

	ACC	Fron_L	Fron_R	Insular_L	Insular_R	Par_L	Par_R
ACC		20/20	20/20	20/20	20/20	0/20	0/20
Fron_L			20/20	20/20	2/20	14/20	0/20
Fron_R				1/20	20/20	0/20	20/20
Insular_L					2/20	20/20	0/20
Insular_R						0/20	20/20
Par_L							20/20
Par_R							

Supplementary Table 2. The estimated parameters of the double gamma function and the measurements of the fitted curves

Parameters	ACC to Fron_R	ACC to Insular_L	ACC to Insular_R	Fron_L to Fron_R	Fron_L to Insular_L	Fron_R to Insular_R	Insular_R to Par_R	Par_L to Par_R
c1	2.53	0.55	2.25	1.16	0.45	5.00	0.96	1.04
a1	7	5	7	7	5	9	3	3
b1	0.96	5.00	1.00	1.17	5.00	0.87	3.23	3.93
t1	1.34	2.69	1.52	1.90	2.91	1.36	1.67	1.32
c2	0.93	0.20	1.01	0.22	0.13	3.61	0.46	0.66
a2	3	10	2	3	10	1	3	3
b2	1.65	2.06	2.97	3.86	2.13	5.00	4.67	5.00
t2	0	0	0	0	0	0	0	0
Measurements								
TTP (s)	8.93	9.36	9.00	9.71	9.92	9.57	8.86	9.78
Magnitude (a.u.)	1.01	0.93	1.02	0.91	0.77	0.92	0.81	0.57
AUC (a.u.)	280.05	237.79	248.18	149.20	295.94	220.79	249.15	124.32
