# Supplementary material for: Characterization of the hemodynamic response function in white matter tracts for event-related fMRI
Source: Nat Commun. 2019 Mar 8;10:1140. doi: 10.1038/s41467-019-09076-2 (PMC6408456; doi:10.1038/s41467-019-09076-2)
Supplement: Supplementary file 4 — Reporting Summary [file 41467_2019_9076_MOESM4_ESM.pdf]

natureresearch

Corresponding author(s): DBBR

Reporting Summary

Nature Research wishes to improve the reproducibility of the work that we publish. This form provides structure for consistency and transparency in reporting. For further information on Nature Research policies, see Authors & Referees and the Editorial Policy Checklist.

Statistical parameters

When statistical analyses are reported, confirm that the following items are present in the relevant location (e.g. figure legend, table legend, main text, or Methods section).

n/a	Confirmed	
☐	☒	The exact sample size (*n*) for each experimental group/condition, given as a discrete number and unit of measurement
☒	☐	An indication of whether measurements were taken from distinct samples or whether the same sample was measured repeatedly
☐	☒	The statistical test(s) used AND whether they are one- or two-sided<br>*Only common tests should be described solely by name; describe more complex techniques in the Methods section.*
☒	☐	A description of all covariates tested
☐	☒	A description of any assumptions or corrections, such as tests of normality and adjustment for multiple comparisons
☐	☒	A full description of the statistics including central tendency (e.g. means) or other basic estimates (e.g. regression coefficient) AND variation (e.g. standard deviation) or associated estimates of uncertainty (e.g. confidence intervals)
☐	☒	For null hypothesis testing, the test statistic (e.g. *F*, *t*, *r*) with confidence intervals, effect sizes, degrees of freedom and *P* value noted<br>*Give P values as exact values whenever suitable.*
☒	☐	For Bayesian analysis, information on the choice of priors and Markov chain Monte Carlo settings
☒	☐	For hierarchical and complex designs, identification of the appropriate level for tests and full reporting of outcomes
☐	☒	Estimates of effect sizes (e.g. Cohen's *d*, Pearson's *r*), indicating how they were calculated
☒	☐	Clearly defined error bars<br>*State explicitly what error bars represent (e.g. SD, SE, CI)*

Our web collection on statistics for biologists may be useful.

Software and code

Policy information about availability of computer code

Data collection	Philips Achieva 3.0T SoftwareVersion 5.3.0 was used to aquire the images. E-prime 2.0(Psychology Software Tools, Inc) was used for presenting the visual stimuli and receiving feedback from the subjects.
Data analysis	The study was developed based on Matlab R2016b (MathWorks, Inc) using a combination of freely available tools that listed below and custom code.<br>SPM12, https://www.fil.ion.ucl.ac.uk/spm/software/spm12/<br>REST V1.8, http://restfmri.net/forum/index.php<br>DTIstudio V3.03 and Diffeomap V1.9, https://www.mristudio.org/

For manuscripts utilizing custom algorithms or software that are central to the research but not yet described in published literature, software must be made available to editors/reviewers upon request. We strongly encourage code deposition in a community repository (e.g. GitHub). See the Nature Research guidelines for submitting code & software for further information.

Data

Policy information about availability of data

All manuscripts must include a data availability statement. This statement should provide the following information, where applicable:

- Accession codes, unique identifiers, or web links for publicly available datasets
- A list of figures that have associated raw data
- A description of any restrictions on data availability

The datasets that generated Fig. 3 - Fig. 7, Supplementary Fig. 1 - Fig. 4 are available in the figshare repository, https://doi.org/10.6084/m9.figshare.7451015.v2.

Field-specific reporting

Please select the best fit for your research. If you are not sure, read the appropriate sections before making your selection.

☒ Life sciences ☐ Behavioural & social sciences ☐ Ecological, evolutionary & environmental sciences

For a reference copy of the document with all sections, see nature.com/authors/policies/ReportingSummary-flat.pdf

Life sciences study design

All studies must disclose on these points even when the disclosure is negative.

Sample size	Twenty individuals were included in the study. No sample size calculation was performed. In our previous study, we observed clear task-specific HRFs in gray matter based on analysis of the time course of fMRI collected from nineteen subjects. We believe that twenty individuals are sufficient for the current study that was based on the same task and similar analysis.
Data exclusions	No data was excluded.
Replication	All attempts at replication were successful.
Randomization	Participants were not allocated into experimental groups.
Blinding	Blinding was not relevant to our study since no subjective evaluation was performed.

Reporting for specific materials, systems and methods

Materials & experimental systems

n/a	Involved in the study	
☒	☐	Unique biological materials
☒	☐	Antibodies
☒	☐	Eukaryotic cell lines
☒	☐	Palaeontology
☒	☐	Animals and other organisms
☐	☒	Human research participants

Methods

n/a	Involved in the study	
☒	☐	ChIP-seq
☒	☐	Flow cytometry
☐	☒	MRI-based neuroimaging

Human research participants

Policy information about studies involving human research participants

Population characteristics	Twenty healthy and right-handed individuals (10M / 10F; age, 29.1 ± 8.8 yrs) with no history of neurological or psychiatric disorders were recruited.
Recruitment	The participants were recruited via flyers placed on campus as well as via personal contacts by the researchers and their associates. Volunteers that are between 21 and 55, and are not having neurological or psychological disorders qualify for this study. There are no biases that are likely to impact the results.

Magnetic resonance imaging

Experimental design

Design type	Event-related.
Design specifications	Twenty-two incongruent words were shown randomly across each session with the interval between any two incongruent words no shorter than 14 s.
Behavioral performance measures	Subjects were instructed to name the color of every word silently as rapidly as possible and provide feedback by clicking a button in response to the color. We recorded the accuracies and response times whose mean ± standard deviation were displayed in results.

Acquisition

Imaging type(s)	Functional, structural, diffusion.
Field strength	3.0
Sequence & imaging parameters	The fMRI images were acquired from these subjects with TR =2 s, TE = 35 ms, SENSE factor = 2, matrix size = 80 × 80, FOV =240 × 240 mm2, 34 slices of 4 mm thickness with a 0.5 mm gap, and 200 dynamics. Diffusion-weighted MR images were acquired using a multi-shot, echo-planar imaging (EPI) sequence with b =1000 s/mm2, 32 diffusion-sensitizing directions, TR = 4.5 s, TE = 84 ms, matrix size=112 × 112 × 68, and voxel size =2 × 2 × 2 mm3. High-resolution T1-weighted images were acquired using a three-dimension (3D) magnetization-prepared rapid gradient-echo (MP-RAGE) sequence at voxel size of 1 × 1 × 1 mm3.
Area of acquisition	Whole brain.

Diffusion MRI ☒ Used ☐ Not used

Parameters: Diffusion-weighted MR images were acquired using a multi-shot, echo-planar imaging (EPI) sequence with b =1000 s/mm2, 32 diffusion-sensitizing directions, TR = 4.5 s, TE = 84 ms, matrix size=112 × 112 × 68, and voxel size =2 × 2 × 2 mm3.

Preprocessing

Preprocessing software	SPM12, REST V1.8, DTIstudio V3.03 and Diffeomap V1.9
Normalization	Images were first linearly co-registered to their T1 and were then non-linearly normalized to a standard space (Montreal Neurological Institute (MNI)) coordinates, at a voxel size of 3 × 3 × 3 mm3.
Normalization template	MNI 152.
Noise and artifact removal	Confounding effects of physiological fluctuations, such as cardiac pulsations and respiration-induced modulations, on fMRI time-series were removed using a CompCor approach.
Volume censoring	N/A

Statistical modeling & inference

Model type and settings	The study was based on the mass-univariate approach. The group analysis was based on a random-effects model.
Effect(s) tested	We studied the effect that reflected the signal changes of incongruent events compared with that of the congruent events. No ANOVA or factorial designs were used.

Specify type of analysis: ☒ Whole brain ☐ ROI-based ☐ Both

Statistic type for inference (See Eklund et al. 2016)	Activated voxel clusters were reported at a threshold p<0.05 (cluster level, family-wise error rate (FWE) corrected) with the cluster size larger than 50 voxels.
Correction	FWE.

Models & analysis

n/a	Involved in the study
☒	☐ Functional and/or effective connectivity
☒	☐ Graph analysis
☒	☐ Multivariate modeling or predictive analysis
